# Editorial: Facial Expression Recognition and Computing: An Interdisciplinary Perspective

**DOI:** 10.3389/fpsyg.2022.940630

**Published:** 2022-05-31

**Authors:** Ke Zhao, Tong Chen, Liming Chen, Xiaolan Fu, Hongying Meng, Moi Hoon Yap, Jiajin Yuan, Adrian K. Davison

**Affiliations:** ^1^State Key Laboratory of Brain and Cognitive Science, Institute of Psychology, Chinese Academy of Sciences, Beijing, China; ^2^University of Chinese Academy of Sciences, Beijing, China; ^3^School of Electronic and Information Engineering, Southwest University, Chongqing, China; ^4^Université de Lyon, CNRS, École Centrale de Lyon LIRISUMR5205, Lyon, France; ^5^Department of Electronic and Electrical Engineering, Brunel University London, London, United Kingdom; ^6^Department of Computing and Mathematics, Faculty of Science and Engineering, Manchester Metropolitan University, Manchester, United Kingdom; ^7^Institute of Brain and Psychological Sciences, Sichuan Normal University, Chengdu, China; ^8^Division of Musculoskeletal and Dermatological Sciences, Faculty of Biology, Medicine and Health, The University of Manchester, Manchester, United Kingdom

**Keywords:** facial expression, recognition, emotion, machine recognition, artificial intelligence

Through the configuration of facial muscles, facial expressions are assumed to reflect a person's internal feelings, emotions, motives, and needs (Ekman et al., 1972). Facial expression recognition plays a crucial role in social interaction. It has been extensively studied in the fields of psychology and artificial intelligence (Russell, 1994; Corneanu et al., 2016; Wood et al., 2016; Liu et al., 2021). The overall goal of this Research Topic was trying to build bridges between human and machine recognition. On the one hand, we provided the latest developments in facial expression recognition, aiming to further understand the cognitive mechanism of how human processes expressions. On the other hand, we collected some studies that use artificial intelligence technology to recognize these expressions.

The first denominator of the collected papers is the attempt to report the latest development in recognizing facial expressions. Balconi and Fronda provided a new perspective to induce and recognize facial expression of emotions based on autobiographic memories rather than emotional movies or pictures. They proposed three steps for creating a database through recalling past autobiographical events of emotional memory. The first step requires collecting individual experiences through mnemonic recall using semi-structured interviews of autobiographical events. The second step requires creating specific algorithms for encoding autobiographical memories. The third step requires the encoding emotional experiences in a personalized linguistic way. Besides, another research conducted by Zhang et al. systematically explored a recognition process for emotional cartoon expressions (happy, sad, and neutral) and the influence of key facial features (mouth, eyes, and eyebrows) on emotion recognition. Qu et al. explored the relationship between facial expressions and time perception.

In addition, a good way to understand the cognitive mechanism of facial expressions processing is through comparison between mental disease and healthy adults. Ma, Guo, et al. investigated the relationship between facial expression recognition and cognitive ability in patients with depression. The results demonstrated that the performance of facial expression recognition is related to the decline of cognitive function, especially for negative emotion. Another study also conducted by Ma, Zhao, et al. found that patients with unipolar depression (UD) had lower performance in recognizing negative expressions, whilst bipolar disorder (BD) had lower accuracy in recognizing positive expressions. Mo et al. explored the confusion effects between depressive patients and healthy controls. Participants were asked to classify each facial expression in a two-alternative forced choice paradigm. Results showed that depressive patients were more inclined to confuse a negative emotion (i.e., anger and disgust) with other expression. Hyniewska et al. found the difference in recognizing facial expression between borderline personality disorder (BPD) and healthy adults. Their results manifested that the ability of emotion recognition in BPD patients was as good as that in healthy individuals, except for the contempt, which were recognized more accurately by BPD patients.

The second denominator is the attempt to report the latest development of artificial intelligence in this domain. Pereira et al. investigated whether existing emotion recognition technology could detect social signals in media interview. Non-verbal signals including facial expression, hand gestures, vocal behavior, and honest signals were captured. The interviews were divided into effective and poor communication exemplars according to the trainers and neutral observers. The correlation-based feature selection method was employed to locate the best feature combination. Naive Bayes analysis produced the best recognition results. Rodríguez-Fuertes et al. analyzed the facial expression of Spanish political candidates in the elections by the algorithms provided in the AFFDEX platform. The basic emotions of each politician were identified and compared through the facial expression analysis. The speech topics associated to the emotions were also identified. Whether there were differences shown by each candidate in every emotion was investigated as well. Namba examined how the feedback of facial expressions affected learning tasks. Learning rate for facial expression feedback was lower than that for symbolic feedback, while no difference between two conditions was found in deck selection or computational model parameters, and no correlation between task indicators and the results of depressive questionnaires was reported.

## Author Contributions

All authors listed have made a substantial, direct, and intellectual contribution to the work and approved it for publication.

## Funding

The paper has been funded by National Natural Science Foundation of China (62061136001 and 32071055) and National Social Science Foundation of China (19ZDA363).

## Conflict of Interest

The authors declare that the research was conducted in the absence of any commercial or financial relationships that could be construed as a potential conflict of interest.

## Publisher's Note

All claims expressed in this article are solely those of the authors and do not necessarily represent those of their affiliated organizations, or those of the publisher, the editors and the reviewers. Any product that may be evaluated in this article, or claim that may be made by its manufacturer, is not guaranteed or endorsed by the publisher.
